# Formative Evaluation and Three-Month Follow-Up of an Online Personalized Assessment Feedback Intervention for Problem Drinkers

**DOI:** 10.2196/jmir.8.2.e5

**Published:** 2006-04-12

**Authors:** John A Cunningham, Keith Humphreys, Kypros Kypri, Trevor van Mierlo

**Affiliations:** ^6^Van Mierlo Communications Consulting IncTorontoONCanada; ^5^School of Medical Practice and Population HealthUniversity of NewcastleAdamstown HeightsNSWAustralia; ^4^Stanford University School of MedicineStanfordCAUSA; ^3^Program Evaluation and Resource CenterVeterans Affairs Health Care SystemStanfordCAUSA; ^2^University of TorontoTorontoONCanada; ^1^Centre for Addiction and Mental HealthTorontoONCanada

**Keywords:** Alcohol, Web-based, self-help, Internet

## Abstract

**Background:**

In recent years, online services for problem drinkers have been developed. This paper describes ongoing efforts to improve one of these services, the Alcohol Help Center.

**Objective:**

This report summarizes new modules added to the Check Your Drinking (CYD) screener, a component of the Alcohol Help Center, to make the CYD screener more useful to periodic heavy drinkers, as well as to regular alcohol consumers. Participants’ initial reactions to the CYD screener and the changes in their drinking habits at a three-month follow-up are presented.

**Methods:**

The CYD screener provides a free personalized Final Report that compares the user’s drinking to that of others in the general population of the same age, gender, and country of origin. Current alcohol consumption and demographic characteristics are collected as part of the CYD screening process. After users were presented with a customized Final Report, they were hot-linked to a volunteer feedback survey. The voluntary feedback survey asked about impressions of the CYD Final Report. Respondents agreeing to participate were sent a follow-up survey after three months.

**Results:**

We recruited 388 volunteers (69% female) who were registered users of another free-to-consumer online eHealth service. Of the 343 respondents agreeing to participate in the three-month follow-up, 138 accessed the survey, and 97 provided complete data (participation rate = 40%; completion rate = 70%). Compared to moderate drinkers, current problem drinkers judged the Final Report to be more useful (34% vs. 69%, χ^2^_1_ = 41.5, *P* < .001) and accurate (43% vs. 76%, χ^2^_1_ = 36.0, *P* < .001). Respondents who participated in the three-month follow-up displayed reductions in drinking compared to baseline (F_4,76_ = 12.2, *P* = .001).

**Conclusions:**

Improvements can still be made to make the CYD screener more relevant to specific populations, particularly periodic heavy drinkers. There is a need to further tailor algorithms that can present questions only relevant to specific populations. There also appears to be a need to further customize the Final Report for respondents who identify themselves as infrequent heavy drinkers. These improvements will be made, and a randomized controlled trial is planned to conduct a rigorous evaluation of the CYD screener as an intervention to help problem drinkers.

## Introduction

Hazardous alcohol consumption has been identified as one of the five leading contributors to the global burden of disease, and it results in enormous economic costs [1-7]. Yet very few people with alcohol problems ever seek treatment; the estimated ratio of treated to untreated problem drinkers ranges from 1:3 to 1:14 in Canada and the United States [8,9-11]. This is due, in part, to concerns about stigma and a desire to deal with their concerns on their own [12,13]. If the majority of people with alcohol concerns do not access traditional treatment programs, would brief, anonymous, 24-hour accessible Internet-based services be more appealing to them? Many problem drinkers have an interest in self-help tools to help them evaluate their drinking [14,15]. Problem drinkers have identified computerized interventions as being particularly attractive [16]. Given this interest, and the high level of online access by problem drinkers (75% in a recent survey) [17], providing tools to problem drinkers on the Internet may promote access to help.

There have been a number of reports of online services for problem drinkers (reviewed in [18,19]). Many of these services would benefit from revisions to take into account the demographic characteristics and the feedback of participants, and to take advantage of the increasing options available to provide sophisticated tools for problem drinkers. A case in point is one of the early online tools for problem drinking, Evaluate Your Drinking [20], a program that provided personalized assessment reports to participants. Preliminary research utilizing a survey hot-linked to the participants’ assessment report found that, while reactions to the assessment reports were generally positive, the report was judged to be less useful by infrequent drinkers as compared to frequent drinkers. In order to increase the usefulness of this online feedback tool, a new version of this program, the Check Your Drinking (CYD) screener (part of the Alcohol Help Center) [21], was created. In addition to updating the normative feedback components using the most recent general population data available, the CYD Final Report incorporates new modules that should appeal to infrequent heavy drinkers. This report describes these improvements and summarizes a preliminary evaluation of the updated intervention.

In order to provide a preliminary outcome evaluation of the CYD screener, a three-month follow-up survey was also conducted. Two hypotheses were tested in this outcome evaluation. Hypothesis one predicted that respondents would be drinking less at three months’ follow-up as compared to baseline. Second, previous research has indicated that respondents’ perceived risk might be an important incentive to adopt health protective behaviors (e.g., [22]). Thus, it would be expected that, as people reduce their drinking, their perceptions of the risk associated with their drinking should also be reduced. Hypothesis two predicted that respondents who displayed reductions in their perceived risk of health consequences from drinking would be more likely to have also reduced their drinking from baseline to three months’ follow-up as compared to respondents who reported no reductions or who reported increased ratings of their perceived risk.

## Methods

### Baseline Survey

Recruitment for this pilot study was conducted by an email invitation sent to registered users of a separate free-to-consumer website program, the Stop Smoking Center [23]. A stand-alone version of the CYD screener was posted on a closed-access website that was custom programmed exclusively for this study. Participants were identified by a randomly generated and anonymous unique variable assigned to each registered user of the Stop Smoking Center. Participants could complete the survey only once, their anonymous user ID being automatically blocked after responding to the survey’s final question. To maximize user privacy, cookies were not used. Volunteers who responded to the email solicitation were taken to a Web page that described the purpose of the study. A full copy of the baseline survey is included in Appendix 1. Because respondents were recruited from the Stop Smoking Center, they were first asked some brief questions about their current smoking status (results reported elsewhere [24]) and whether they currently drank alcohol. Those respondents who were current drinkers were asked to complete the CYD screener and receive their personalized Final Report, while those who indicated that they abstained from alcohol consumption were thanked for their participation and were not asked to complete the CYD screener. At the end of the Final Report, respondents were asked if they were willing to participate in a three-month follow-up, and they were provided with a hot-link button that took them to a voluntary survey that asked if they found the Final Report useful (not at all useful; slightly useful; somewhat useful; extremely useful), if anything was surprising in the Final Report (no; surprised how much more drank than others; surprised how much less drank than others; something else surprising), if they felt the Final Report was an accurate summary of their drinking (yes; no, infrequent drinker; no, drinking varies over time), and to what extent they believed they would personally be at risk of getting hurt or sick because of their drinking (0 = no risk; 10 = high risk). Respondents were also provided with pictures of each of the three main drinking summary graphs (see description below) and were asked to place a check mark under the graphs they found useful (or, if they found none useful, to not check any of the graphs). Finally, text boxes were available for respondents to provide written comments, but written comments were not mandatory. Survey items were not presented in random order. The maximum number of survey items was eight on one page, and the survey was distributed over 10 pages. The survey employed client-side and server-side error checking, required field validation, and server-side data validation. Participants could not proceed through the survey until they had responded to all mandatory questions on each page. Although the majority of questions were static and mandatory, some questions requested the participant’s opinion (not mandatory). Until survey completion, participants were able to review and change their answers by clicking the back button on their browser or the back button inserted at the bottom of each survey page.

The study was approved by the standing ethics committee of the Centre for Addiction and Mental Health. The email invitation described the purpose of the survey, how long it would take (about 10 minutes), and that the use and storage of the data would ensure anonymity. Responding to the email invitation was taken as informed consent. The design of the survey followed international guidelines set forth to protect privacy [25,26]. The survey was pre-tested for usability and technical functionality prior to release. Details of the survey research methods have been presented in compliance with the checklist for reporting results of Internet e-surveys (CHERRIES) [27].

### Three-Month Follow-up Survey

The same survey methods were employed for the three-month follow-up survey as for the baseline survey. A full copy of the follow-up survey is available in Appendix 2. First, respondents were asked about their current smoking status, whether they currently drank alcohol, and to what extent they currently perceived themselves to be at risk of getting hurt or sick because of their drinking (1 = no risk; 10 = high risk). Respondents who were current drinkers were then asked the same items from the CYD screener (see below), this time with respect to their drinking in the last three months. Results from the follow-up survey were linked to the baseline survey using respondents’ unique user ID number.

### Statistical Analysis

Univariate comparisons were made of the baseline survey results, comparing respondents who did or did not complete the voluntary feedback survey at baseline and also comparing problem and nonproblem drinkers. A repeated-measures multivariate analysis of variance was employed to test hypotheses one and two. Differences in drinking from baseline to follow-up were compared for respondents who did or did not report reductions in their perceived risk associated with drinking between the baseline and follow-up time points.

### The Check Your Drinking Screener

The CYD screener is available for public access [21]. The survey first asks respondents their gender, age, country of origin, weight, and how much money a drink usually costs them. The respondents are also asked their reason for taking the CYD screener (for yourself; for someone you know; you are just checking out the CYD test to see what the results look like), which provides an option for participants to indicate that they are researchers or health professionals (so researcher data can be removed from the sample). The first page contains a description of the CYD screener with a link to a sample Final Report, and it describes the uses to which the data will be applied. After submitting the first page, respondents complete an 18-item survey that asks about details of their drinking. The screener includes the Alcohol Use Disorders Identification Test (AUDIT) [28,29], a well-validated measure that distinguishes between problem and nonproblem drinkers (cut-off score of eight or more on the AUDIT indicates a current problem with alcohol). Respondents are also asked to estimate how much they drink on each day of a typical week [30,31] and to report the highest number of drinks they consumed on a single occasion. The CYD survey concludes by asking respondents if they have experienced any of six psychosocial consequences as a result of their drinking in the last year: harmful effect on (1) friendships/social life; (2) physical health; (3) home life or marriage; (4) outlook on life (happiness); (5) work, studies, or employment opportunities, or (6) financial position [32].

### Final Report

The Final Report begins with a summary pie chart that compares the respondent’s drinking in a typical week to that of others of the same age group (six different age groups), gender, and country of origin. Recent population comparison data are currently available for Canada and the United States; UK data have been added since this study and data from other countries will be added at a later date [33-36]. The respondents are then provided with an estimate of the percentage of days they drank in the last year, the number of drinks they consumed in the last year, and the greatest number of drinks they consumed on one occasion. To heighten the impact of this customized information, estimates are provided of the amount of money spent on drinking and the number of calories consumed, including an estimate of the amount of weight added in the past year as a result of drinking.

The Final Report then continues with two drinking feedback graphs—a bar chart comparing the respondent’s drinking on each day of the week to that of others of the same age group and gender (data only available from Canada), and a pie chart comparing the frequency of heavy-drinking days (five or more drinks on one occasion) to that of others of the same age, gender, and country of origin. This last graph, in particular, was added with the specific intent to provide useful feedback to infrequent drinkers. Respondents who drink five or more drinks on one occasion once per month or more are alerted to the increased risks associated with this type of consumption [37]. A list of the actual psychosocial consequences the respondent endorsed is also provided. Next, a dose-response chart is presented that describes the chances of experiencing negative consequences as a result of the weekly alcohol consumption (generated using data from the 2004 Canadian Addiction Survey [33]). A chart graphically depicting the respondent’s AUDIT score is also provided along with an explanation of what different AUDIT scores indicate. The Final Report continues with an estimate of the amount of time it takes respondents to metabolize one, four, and ten drinks (based on weight), and it calculates how many hours they were under the influence of alcohol in the past year. The report concludes with sensible drinking guidelines provided by the Centre for Addiction and Mental Health [38], a summary of the health effects of alcohol, and a list of the different things a respondent could do in order to reduce the risks associated with drinking. A complete example of a Final Report can be found in Appendix 3.

## Results

### Baseline Survey

Email invitations were sent out to 7741 registered users of the Stop Smoking Center who registered between October 27, 2004 and July 27, 2005 and had active email accounts. Of these potential participants, 1085 recipients hot-linked to the survey using the unique link provided in each email (participation rate = 14%). Of these, 973 started the baseline survey; 9 respondents were removed because they said they were taking the test for someone else; 231 were removed because they identified themselves as nondrinkers, and 1 respondent did not complete the CYD survey, resulting in a final sample size of 732. Of these 732 respondents, 388 (53%) completed the voluntary feedback survey to give their impressions of the Final Report (completion rate = 40%). Table 1 presents the demographic and drinking characteristics of respondents who completed and respondents who did not complete the voluntary feedback survey. There were no significant differences in any of the demographic or drinking characteristics between survey completers and noncompleters.

**Table 1:** Demographic and drinking variables of users of the Check Your Drinking screener

		**Completed Volunteer Survey**	**Did Not Complete Voluntary Survey**	***P***
		**n = 388**	**n = 344**	
Mean age (years) (SD)		40.3 (11.3)	38.9 (11.8)	.12
Female (%)		68.8	66.6	.57
**Country of Origin**				
	United States (%)	62.6	61.7	
	Canada (%)	18.0	14.0	
	Other (%)	19.3	24.3	.14
Mean number of drinks/typical week (SD)		9.5 (10.9)	10.6 (12.3)	.21
Mean AUDIT score (SD)^*^		7.2 (6.1)	7.4 (5.8)	.61
Mean number of alcohol consequences (SD)^†^		1.2 (1.8)	1.2 (1.8)	.62

^*^Problem drinking defined as a score of eight or more on the Alcohol Use Disorders Identification Test (AUDIT) [28,39]

^†^Has drinking ever affected (1) friendships/social life; (2) physical health; (3) home life or marriage; (4) outlook on life (happiness); (5) work, studies, or employment opportunities; or (6) financial position [32]

**Table 2:** Voluntary feedback survey, comparing problem and nonproblem drinkers

		**NonproblemDrinkers**	Problem Drinkers^*^	***P***
		**n = 258**	**n = 130**	
Mean age (years) (SD)		41.5 (11.8)	37.9 (9.7)	.001
Female (%)		74.0	58.5	.003
**Country of Origin**				
	United States (%)	67.1	53.8	
	Canada (%)	20.2	13.8	
	Other (%)	12.8	32.3	.001
Mean number of drinks/typical week (SD)		4.6 (4.6)	19.1 (13.2)	.001
Mean number of alcohol consequences (SD)^†^		0.3 (0.8)	2.8 (2.1)	.001
Mean perceived risk (SD)^‡^		0.7 (1.0)	4.7 (3.0)	.001
**Impressions of Feedback**				
Feedback somewhat/extremely useful (%)		34.1	69.2	.001
Surprised how much more drink than others (%)		13.6	50.0	.001
Summary captures drinking (%)		43.4	76.2	.001
Typical week graph useful (%)		28.7	42.3	.01
Days of week summary useful (%)		23.6	38.5	.003
Frequency 5+ drinks graph useful (%)		13.6	16.2	.60

^*^Problem drinking defined as a score of eight or more on the Alcohol Use Disorders Identification Test (AUDIT) [28,39]

^†^Has drinking ever affected (1) friendships/social life; (2) physical health; (3) home life or marriage; (4) outlook on life (happiness); (5) work, studies, or employment opportunities; or (6) financial position [32]

^‡^To what extent do you believe that you are personally at risk of getting hurt or getting sick because of your own drinking (0 = no risk; 10 = high risk) [40]

Table 2 presents demographic and drinking characteristics and impressions of the Final Report for problem drinkers (defined as an AUDIT score of eight or more) and moderate drinkers who completed the voluntary feedback survey. Problem drinkers were younger (*t* = 3.2, *P* = .001) and more likely to be male (χ^2^_1_ = 9.1, *P* = .003) compared to current moderate drinkers. Problem drinkers were also more likely than moderate drinkers to live outside the United States or Canada (χ^2^_2_ = 21.3, *P* < .001). Further inspection of the country of origin revealed that 48% of the respondents who lived outside of the United States or Canada lived in the United Kingdom. As expected, problem drinkers consumed more alcohol in a typical week (*t* = 12.2, *P* < .001) and experienced more drinking consequences (*t* = 12.8, *P* < .001) compared to moderate drinkers. Problem drinkers also rated themselves as significantly more likely to get hurt or sick because of their drinking compared to moderate drinkers (*t* = 14.7, *P* < .001).

There were also a number of significant differences regarding impressions of the Final Report between problem and moderate drinkers (see Table 2). Problem drinkers were more likely to find the feedback summary somewhat or extremely useful (χ^2^_1_ = 41.5, < .001), to be surprised by how much more they drank than others (χ^2^_1_ = 58.1, *P* < .001), and to feel that the summary accurately outlined and captured their drinking (χ^2^_1_ = 36.0, *P* < .001). Because one of the main intents of updating the screener was to provide useful information for infrequent drinkers, two further analyses were conducted comparing problem drinkers who were frequent or infrequent drinkers. Compared to problem drinkers who drank more than once a week (n = 103), those who drank weekly or less (n = 27) appeared just as likely to find the Final Report somewhat or extremely useful (69.9% vs. 66.7%, χ^2^_1_= .008, *P* = .93). In addition, problem drinkers who consumed five or more drinks (on one occasion) once a month or more (n = 111) were just as likely to find the Final Report somewhat or extremely useful as those problem drinkers who consumed five or more drinks less than once a month (n = 19, 69.4% vs. 68.4%, χ^2^_1_ = .001, *P* = 1.0). There was some difference in the proportion of frequent (more than weekly) and infrequent (weekly or less) problem drinkers who thought the feedback accurately depicted their drinking (81.6% vs. 55.6%, χ^2^_1_= 6.6, *P* = .01). However, there was no difference between frequent heavy drinkers (five or more drinks monthly or more) and infrequent heavy drinkers on how accurate they felt the Final Report to be (75.7% vs. 78.9%, χ^2^_1_ = .001, *P* = .99).

Respondents were asked if they found the three drinking feedback charts useful (see Table 2). Compared to moderate drinkers, problem drinkers more often found the weekly drinking pie chart (χ^2^_1_ = 6.6, *P* = .01) and the days of the week drinking bar chart useful (χ^2^_1_ = 8.6, *P* = .003). Few problem or moderate drinkers found the frequency of heavy-drinking days pie chart useful (χ^2^_1_= .28, *P* = .60). One potential difficulty in interpreting respondents’ ratings was that the feedback charts were generated with population data from Canada or the United States, so they would be less relevant to respondents from other countries. Analyses were conducted to explore the proportions of Canadians and Americans who endorsed each chart and were marginally higher than those reported by the full sample (not shown).

### Three-Month Follow-Up Survey

Of the 343 respondents who agreed to participate in the three-month follow-up survey, 138 accessed the survey and attempted to provide responses (participation rate = 40%). Responses from 41 participants could not be used because the unique respondent ID number was not associated with the participants’ data. (The email invitation to participate in the three-month follow-up contained a link to the follow-up survey that was unique to the participant. Depending on the size of the participant’s email window, this link could extend over more than one line. Respondents whose link extended over more than one line were able to access the survey, but their unique ID number was not associated with their responses, making the data unusable.) This left 97 participants who provided complete follow-up data (completion rate = 70%). Finally, 16 of these respondents did not complete the baseline voluntary feedback survey and, as such, had not provided an assessment of perceived risk at baseline, leaving 81 respondents with complete data to test hypothesis two. A repeated-measures multivariate analysis of variance (MANOVA) was conducted to test both hypotheses one and two simultaneously. The two independent variables were time (baseline versus three-month follow-up) and change in perception of risk (reduction in perception of risk from baseline to follow-up versus no reduction or increase in perceived risk). Four drinking variables were included as dependent variables: number of drinks in a typical week, greatest amount drunk on one occasion, number of drinking related consequences, and AUDIT score. Baseline and follow-up values for these dependent variables are displayed in Table 3. The MANOVA revealed a main effect of time (F_4,76_ = 12.2, *P* = .001) and of reduction in perceived risk (F_4,76_ = 5.3, *P* = .001). In addition, there was a significant interaction between time and perceived risk (F_4,76_ = 6.1, *P* = .001). Subsequent univariate analyses exploring this interaction revealed significant interactions for the variables: number of drinks in a typical week (F_1,79_ = 4.0, *P* = .05), greatest amount drunk on one occasion (F_1,79_ = 6.1, *P* = .02), number of drinking-related consequences (F_1,79_ = 24.5, *P* = .001), and AUDIT scores (F_1,79_ = 5.4, *P* = .02). Inspection of the observed means for these variables revealed that respondents who had a reduction in their perceived risk from baseline to follow-up also had reductions in their drinking from baseline to follow-up. Respondents with no reduction or an increase in their perceived risk displayed little or no reductions in their drinking from baseline to follow-up.

**Table 3:** Mean alcohol consumption at baseline and three-month follow-up by reduction in perceived risk from baseline to follow-up

	Reduction in Risk^*^n = 24		**No Reduction in Riskn = 57**		
	**Baseline**	**Follow-Up**	**Baseline**		**Follow-Up**
Mean number of drinks/typical week (SD)	16.7 (10.8)	14.5 (12.2)	8.1 (11.0)		8.8 (12.9)
Mean greatest amount drank (SD)	9.3 (3.5)	6.0 (3.4)	6.4 (4.8)		5.1 (5.3)
Mean number of alcohol consequences (SD)	3.2 (2.1)	1.5 (1.9)	0.9 (1.7)		0.7 (1.6)
Mean AUDIT score (SD)	13.1 (6.5)	11.1 (6.4)	6.0 (6.1)		5.6 (6.3)

^*^These respondents rated their perceived risk of drinking as less at the three-month follow-up than at baseline (right after receiving their Final Report).

## Discussion

### Principal Results

Problem drinkers were more likely to find the Final Report useful, surprising, and accurate than moderate drinkers. As the primary target of this website is current problem drinkers, it was intended that the Final Report should be found most useful to this group (although attempts were made to make the Final Report relevant to respondents from the entire continuum of alcohol consumption, from social drinkers to those dependent on alcohol). Of the feedback elements in the Final Report, both the original typical week pie chart and the days of the week bar chart were endorsed by about a third of respondents as being useful. The frequency of five or more drinking days pie chart was not often endorsed as being useful. It was discouraging to see how few respondents found the frequency of five or more drinks pie chart useful. This element of the CYD screener was added specifically to make the Final Report more relevant to infrequent heavy drinkers. Some qualitative responses from participants also highlighted that the Final Report was considered inaccurate by irregular drinkers.

As with an evaluation of the earlier version of the CYD [20], a significant proportion of respondents were female. One of the potential advantages of online services is the ability to reach groups of people (such as females) who are less likely to seek help from traditional services. Also similar to the earlier report was the proportion of respondents who were current problem drinkers. This is despite the fact that the recruitment method for the current evaluation was unusual—an invitation to current users of an online tobacco cessation self-help service. While an excellent means of quickly recruiting a large sample to a new online service (recruitment period was one week), caution should be taken in assuming that these respondents have the same profile as those who will find the Alcohol Help Center on their own.

There was a significant reduction in drinking measures from baseline to follow-up. While this finding supports hypothesis one, it should be stressed that this finding does not confirm that the reduction in drinking was due to use of the CYD screener because there was no control group in this study. In addition, reductions in estimates of perceived risk from baseline to follow-up were associated with reduction in drinking. This finding provides support for the importance of perceived risk as a potential mediator of the impact of self-help interventions such as the CYD screener. However, as with the preliminary support that the CYD may lead to reductions in drinking, a proper randomized controlled trial is needed in order to confirm this hypothesis [41].

### Limitations

Not all respondents filled out the voluntary feedback survey, suggesting that caution should be taken regarding the generalizability of the results. It should, however, be noted that there were no systematic differences between completers and noncompleters on the variables we measured. In addition, there was a substantial attrition of respondents from baseline to follow-up, again leading to cautions regarding the validity of the results [42,43]. Finally, the present study was not a randomized controlled trial, so observations of reductions in drinking can only be taken as peripheral support for the effectiveness of this online intervention.

### Future Directions

An upgraded version of the CYD screener will include a modified assessment algorithm and Final Report for participants with irregular drinking patterns rather than using the same assessment and Final Report for all users. Finally, a randomized controlled trial is underway to establish whether participation in the CYD screener will result in sustained reductions in alcohol consumption.

## Multimedia Appendix 1

Baseline survey.

## Multimedia Appendix 2

Follow-up survey.

## Multimedia Appendix 3

Sample Final Report.

## Abbreviations

AUDIT: Alcohol Use Disorders Identification Test
CYD: Check Your Drinking
