# Supplementary material for: Formative Evaluation and Three-Month Follow-Up of an Online Personalized Assessment Feedback Intervention for Problem Drinkers
Source: J Med Internet Res. 2006 Apr 12;8(2):e5. doi: 10.2196/jmir.8.2.e5 (PMC1550700; doi:10.2196/jmir.8.2.e5)
Supplement: Supplementary file 1 [file jmir_v8i2e5_app1.pdf]

Survey One Screen Grabs

File Name: Survey 1 Email Invitation

CHECK YOUR DRINKING

The STOP SMOKING CENTER

Dear SSC Member,

In the past you signed up for the free Online Support Group and Quit Program at www.StopSmokingCenter.net.

We're inviting our members to complete an anonymous online survey. It should only take about 10 minutes to complete and you'll get personalized feedback on your drinking patterns (although we also encourage non-drinkers to participate as well).

The purpose of the survey is to help us gain a better understanding about the relationship between smoking and drinking alcohol. The results will help our team learn how we can do a better job at helping people quit and how they respond to "triggers" that might cause them to start smoking again.

The survey also has a research purpose. The research team will publish the results in both a scientific journal and an international meeting so others can become better aware of our free programs.

To access the survey please click on the link below (If the below link is displayed on two lines please copy and paste the full link into your web browser):

http://dev.checkyourdrinking.net/survey/ss1.aspx?id=NDqyMA%3d%3d-KjspLuWSC%2bU%3d

It's important for us tell you that the survey is completely anonymous: we have no way of finding out who you are, where you live, we can't link the survey to your email address and we can't link it to any personal information. The answers to the questionnaire are cumulative, which means that we'll group your answers with the thousands of others who also answer the survey.

If you have any questions about this survey please feel free to write our Support Staff or by contact our Privacy Officer by writing to privacy@v-cc.com.

Thanks in advance for helping us help others!

Most Sincerely,

The Stop Smoking Center Support Team

The Stop Smoking Center is for educational purposes only and is not to replace the advice of your GP or other health care provider. SSC Version 5.0 is Copyright 2005 by V-CC. All rights reserved including related methods and software. All worldwide patent rights reserved.

File Name: Survey 1 Page 1

CHECK YOUR DRINKING

The STOP SMOKING CENTER

Thanks for helping the Stop Smoking Center!

In this anonymous survey we're going to first ask you questions about smoking, and then we'll ask you some questions about drinking alcohol. When you're finished the survey we'll give you a Final Report. The Final Report will give you a comparison of your drinking to other people the same age and sex. It should take no more than about 10 minutes to complete this survey.

After you've received and read your Final Report we're going to ask you a few feedback questions. We're grateful to your feedback so we can improve the program.

Let's get started with some questions about your current or past smoking:

1. At present do you smoke daily, occasionally, or not at all?

- ○ Daily
- ○ Occasionally
- ○ Not at all

Back Next

File Name: Survey 1 Page 2

CHECK YOUR DRINKING

The STOP SMOKING CENTER

1. How long ago was it that you last smoked?

- Less than one week
- More than one week but less than a month
- 1 to 6 months
- 7 to 11 months
- 1 to 5 years
- More than 5 years

2. Before you quit, how many cigarettes did you usually smoke each day?

3. When you drink alcohol, do you ever experience a strong urge, desire or thoughts about smoking?

- I don't drink alcohol
- Never
- Occasionally
- Frequently
- All the time

4. When you used to smoke, did you ever experience a strong urge, desire or thoughts about drinking alcohol?

- Never
- Occasionally
- Frequently
- All the time
- Only when I was around people who were drinking

Back Next

File Name: Survey 1 Page 3

CHECK YOUR DRINKING

The STOP SMOKING CENTER

How many drinks do you normally have before you experience strong urges, desires or thoughts about smoking?

How many drinks do you normally have before you experience these strong urges, desires or thoughts about smoking?

You are: ○ male ○ female

Your date of birth: --------------- -------------------- ---

What country do you live in?

How much do you weigh?

You are taking this test:

○ For yourself

○ For someone you know

○ You are just checking out the CYD test to see what the results look like

How much would you say that each drink costs you? (example: 3.75)

Back Next

File Name: Survey 1 Page 4

CHECK YOUR DRINKING

One Standard Drink Equals...

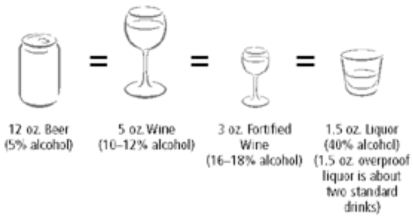

1. How often do you have a drink that contains alcohol?
- ○ never
- ○ monthly or less
- ○ 2-4 times a month
- ○ 2-3 times a week
- ○ 4 or more times a week

2. On a typical day when you do drink, how many drinks containing alcohol do you have?
- ○ 0-2
- ○ 3-4
- ○ 5-6
- ○ 7-9
- ○ 10+

3. How often do you have 5 (five) or more drinks on one occasion?
- ○ never
- ○ less than monthly
- ○ once per month
- ○ 2-3 times per month
- ○ weekly
- ○ 2-4 times per week
- ○ daily or almost daily

4. How often during the last year have you found that you weren't able to stop drinking once you started?
- ○ never
- ○ less than monthly
- ○ monthly
- ○ weekly
- ○ daily or almost daily

5. How often during the last year have you failed to do what's normally expected from you because of drinking?
- ○ never
- ○ less than monthly
- ○ monthly
- ○ weekly
- ○ daily or almost daily

Back | Next

CheckYourDrinking.net is for educational purposes only and is not to replace the advice of a healthcare professional.
Copyright 2001-5 by V-CC. All Rights Reserved. All worldwide patent rights reserved.

File Name: Survey 1 Page 5

6. How often during the last year have you needed a first drink in the morning to "get yourself going" after a heavy drinking session?

- never
- less than monthly
- monthly
- weekly
- daily or almost daily

7. How often during the last year have you had a feeling of guilt or remorse after drinking?

- never
- less than monthly
- monthly
- weekly
- daily or almost daily

8. How often during the last year have you been unable to remember what happened the night before because you had been drinking?

- never
- less than monthly
- monthly
- weekly
- daily or almost daily

9. Have you or someone else been injured as a result of your drinking?

- no
- yes, but not within the last year
- yes, during the last year

10. Has a relative or friend or doctor or other health professional ever shown concern about your drinking, or suggested that you cut down?

- no
- yes, but not within the last year
- yes, during the last year

Back Next

CheckYourDrinking.net is for educational purposes only and is not to replace the advice of a healthcare professional.
Copyright 2001-5 by V-CC. All Rights Reserved. All worldwide patent rights reserved.

File Name: Survey 1 Page 6

CHECK YOUR DRINKING

One Standard Drink Equals...

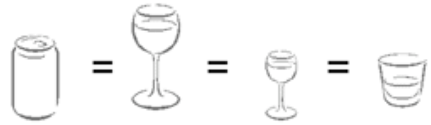

12 oz. Beer (5% alcohol) = 5 oz. Wine (10–12% alcohol) = 3 oz. Fortified Wine (16–18% alcohol) = 1.5 oz. Liquor (40% alcohol) (1.5 oz. overproof liquor is about two standard drinks)

11. What was your drinking like during a typical week in the last year (12 months)? We realize that this will only be a rough estimate, but please indicate the approximate number of drinks you usually drank on each day of the week.

MON	TUES	WED	THURS	FRI	SAT	SUN
—	—	—	—	—	—	—

12. What is the greatest number of drinks you've had on one day in the past 12 months?

—

Back Next

CheckYourDrinking.net is for educational purposes only and is not to replace the advice of a healthcare professional.
Copyright 2001-5 by V-CC. All Rights Reserved. All worldwide patent rights reserved.

File Name: Survey 1 Page 7

CHECK YOUR DRINKING

13. Within the last twelve (12) months was there ever a time that you felt your alcohol use had a harmful effect on your friendships or social life?

- no
- yes

14. Within the last twelve (12) months was there ever a time that you felt your alcohol use had a harmful effect on your physical health?

- no
- yes

15. Within the last twelve (12) months was there ever a time that you felt your alcohol use had a harmful effect on your outlook on life (happiness)?

- no
- yes

16. Within the last twelve (12) months was there ever a time that you felt your alcohol use had a harmful effect on your home life or marriage?

- no
- yes

17. Within the last twelve (12) months was there ever a time that you felt your alcohol use had a harmful effect on your work, studies or employment opportunities?

- no
- yes

18. Within the last twelve (12) months was there ever a time that you felt your alcohol use had a harmful effect on your financial position?

- no
- yes

Back | Click Here to Generate Your Final Report

CheckYourDrinking.net is for educational purposes only and is not to replace the advice of a healthcare professional.
Copyright 2001-5 by V-CC. All Rights Reserved. All worldwide patent rights reserved.

File Name: Survey 1 Final Report

Here is your Final Report. When you're finished reviewing or printing your Final Report please click "Next" at the bottom left of your screen so we can ask you a few questions about the Final Report. Your answers will help us improve the Final Report and your feedback is very important to us!

Click here for a printer friendly version of your Final Report

Your Final Report

The average number of drinks you reported consuming per week was 14.

How do you compare to males your age from Canada? The highlighted slice of the pie chart below is where your drinking fits compares to other males in your age range from Canada.

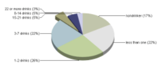

Within the last twelve (12) months:

- You reported drinking on approximately 42.7% of days in the last year.
- You reported that you drank a total of 728 drinks in the last year.

This also means that:

- You spent approximately $3640 in the last year, depending on where you drank (at home, in a bar, etc.).
- You consumed (on average) 400 calories from alcohol on days that you drink. Based on the total amount of drinking you had enough alcohol to add roughly 21 pounds to your weight in the last year. **Note:** One drink has about 100 calories and 3,500 calories roughly equals 1 extra pound of weight.
- You also reported that within the past year, the greatest number of drinks you had on one occasion was 12 drinks.

Your Drinking Patterns

The following graph outlines how your weekly alcohol consumption rates compare to other males in your age range from Canada.

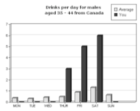

Heavy Drinking Days

How often do males from Canada in your age range drink five or more drinks on one occasion? The highlighted slice shows where your drinking fits into the chart:

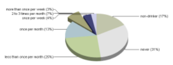

Why care about having five or more drinks on the same day? Heavy drinking days place you at increased risk of experiencing negative consequences because of your drinking. If you got rid of these heavy drinking days you would reduce your chances of experiencing problems by about 50%.

Alcohol-Related Consequences

In the last twelve (12) months you reported your drinking had a harmful effect on your:

- friendships or social life
- physical health
- level of happiness
- home life or marriage
- work, studies or employment opportunities
- financial position

Risky Drinking

A national survey looked at how much people drank in a week and how their drinking might be affecting different areas of their lives. People were asked about their physical health, outlook on life, friends/social life, relationships with their spouse, partner or children, home life, financial position and work or studies. The results showed that the more people drank in a week, the greater the chance that drinking was affecting more and more areas of their lives

How likely are you to have problems as a result of your drinking? The white bar on the chart below shows where you fit:

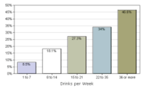

AUDIT Score

The AUDIT questionnaire was developed by the World Health Organisation to evaluate a person's use of alcohol. The AUDIT score shows whether a person's drinking should be considered a problem. Higher scores usually mean serious problems. The below chart is in the shape of a pyramid to show that there are more people with low AUDIT scores than high ones.

Your AUDIT score is 20. Based on the answers that you supplied during the CYD survey, the white area of the chart shows where your score falls.

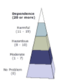

The following describe the meaning of AUDIT scores

Score of	Means
0-7	Low risk of alcohol-related harm
8-10	High risk of experiencing alcohol-related harm (some people in this range will already be experiencing significant harm)
11-19	A person scoring in this range will already be experiencing significant alcohol-related harm.
20+	A person scoring in this range may be alcohol dependent and is advised to see a health care professional about their drinking.

Burning Alcohol

Your liver metabolizes (or burns) alcohol at a constant rate – about 1 gram per hour for every 10 kilograms (22 pounds) of your body weight. Exercising or drinking coffee will not get the alcohol out of your body any sooner.

Although you may not feel some of the effects of alcohol your body is working long after you drink to get rid of the alcohol in your system. Depending on how much you drink and weigh, your liver can be under extra strain for a very long time. This is one example of the health risks of heavy drinking.

You reported that you weigh 185 lbs. This means that:

- If your liver is healthy, burning one drink takes you 2 hours. Burning four drinks takes you about 7 hours. If you had ten drinks, it will take about 17 hours until there is no alcohol in your system.
- In the last year you spent about 1,212 hours under the influence of alcohol (based on your typical drinking during one week).

There are also other kinds of risk that heavy drinking presents. Even small amounts of alcohol can affect your ability to drive or operate heavy equipment safely. **If you have a drink, don't drive!** Take a bus or taxi, or get a lift from a friend who hasn't been drinking.

Sensible Drinking

Guidelines supported by the Centre for Addiction and Mental Health suggest that most people can drink up to two drinks a day without significant risk to their health, in the short or the long term.

Most people can and do drink safely and sensibly. This means no more than two drinks a day with a weekly maximum of 14 drinks for men and 9 drinks for women. It is also a good idea to make sure there are days when you don't drink at all. For some people, even 1 to 2 drinks per day would be too many. Pregnant women, for example, are advised to abstain from alcohol completely because even small amounts of alcohol could increase the risk to the unborn child. Heavy drinking days are especially dangerous to the unborn child. Certain health problems such as heart disease or cancer can make even moderate drinking unsafe.

Health Effects of Alcohol

We've included the following information in the event that you would like to learn more about how the use of alcohol can affect your health.

Your liver
Because the liver receives blood directly from the intestines, it takes the brunt of high alcohol concentrations. Heavy alcohol consumption can lead to two serious types of liver injury: *hepatic inflammation* (alcohol hepatitis) and *progressive liver scarring* (fibrosis or cirrhosis). (*Chedid et al 1991; Dufour et al 1993*)

Women are more at risk to serious alcohol-related liver injury; they develop cirrhosis at a lower dose of alcohol than men do. (*Marbet et al 1987*)

Heavy drinkers are 3 times more likely to develop liver cancer than non-drinkers. (*English & Holman, 1995*)

Your throat, stomach, and intestines
Alcohol is a cause of long-term throat inflammation that sometimes leads to cancer.
Inflammation occurs in part because alcohol reduces contraction of the smooth muscle in the lower throat. (*Keshavarzian et al 1994*)

People who drink more that 21 drinks per week have almost a ten-fold higher risk of throat cancer than those who consume fewer than 7 drinks per week. (*Vaughan et al 1995*)

Mouth cancers are six times more common in heavy alcohol users than in non-alcohol users.
(*American Cancer Society, 2002*)

Your pancreas
The pancreas is a gland behind your stomach that releases chemicals important for digesting food. Heavy alcohol use can lead to long-term pancreatic inflammation, weakening, and scarring. (*Haber et al 1995*)

Alcohol can also cause acute pancreatitis, a severe and very painful inflammation of the pancreas.

Your heart and circulatory system
The potential health benefits of moderate drinking (up to 2 drinks per occasion) do not apply to younger people, whose risk for heart disease is ordinarily very low.

Long-term drinking of more than four drinks per occasion has been linked to a variety of damaging effects on the heart and circulatory system. (*Davidson, 1989*)

Your brain
Alcohol can cause direct or indirect damage to nervous tissues. Long-term heavy drinking is linked to brain damage and poor mental functioning. (*Spreen and Strauss, 1991*)

Your endocrine system (hormone regulation)
Alcohol interferes with the hormone regulation of a number of bodily activities. Men who have a history of heavy drinking often have lower levels of testosterone and increases in female sex steroids, such as estradiol and estrone.

Emergency Department
Patients treated in an emergency department for an unintentional injury are 13.5 times more likely to have drunk 5 or more alcohol-containing drinks within 6 hours of their injury. Alcohol-related unintentional injuries and deaths include motor vehicle crashes, drowning, falls, hypothermia, burns, suicides, and homicides. (*Vinson, 2003*)

Road traffic crashes
Road traffic crashes are the leading cause of death for people aged 1-34 years. They are also a leading cause of hospitalization for serious injury. Alcohol is involved in around 40% of crashes (LTSA, 2000; NHTSA, 2003).

Compared with a person with no alcohol in their blood, a person with a blood alcohol concentration (BAC) of 0.10% is between 13 and 18 times more likely to have any crash and 50-90 times more likely to have a fatal crash. (*Miller, 2001*)

Unlike what some people believe, vehicle occupants with high levels of alcohol in their system (high BAC) are more likely to be seriously injured or to die in the event of a crash. (*Soderstrom, 1993*)

Alcohol and Violence
In 1997, about 40% of all crimes (violent and non-violent) were committed by people who had alcohol in their system. (*Bureau of Justice Statistics, 1998*)

In 1997, 40% of convicted rape and sexual assault offenders said that they were drinking at the time of their crime. (*Greenfield, 2000*)

Approximately 72% of rapes reported on college campuses occur when victims are so intoxicated they are unable to consent or refuse. (*Wecshler, 2004*)

Reducing Your Risk

There are many things that you can do to reduce the risk of hurting yourself or others. Here are some small steps you can take to start making a change:

- Don't drink in any situation where there's a risk of accident or injury — for example, drinking and driving.
- Don't mix alcohol with other drugs — especially other depressants like tranquilizers, barbiturates, heroin or other opioids.
- Try to reduce by one or two days the number of days you drink each week.
- Decide how much you will drink ahead of time and keep yourself to this limit.
- Take a limited amount of money with you if you go out to have a drink.
- Keep track of the amount you drink.
- Alternate alcoholic with non-alcoholic beverages when you drink.
- Choose alcoholic beverages with lower alcohol content.
- If you are out drinking with friends, make sure at least one person stays sober. If that person is driving they should not drink any alcohol at all.
- Do not become intoxicated with people you do not know and trust; criminal victimization is a much greater risk when you are drunk.
- Remember the need to practice safer sex — always use condoms.

We're going to review the answers that Stop Smoking Center members gave us, and we're going to send a brief follow-up survey in three months time. Would you like to help us out by receiving an invitation to complete the follow up survey in three months?

○ **Yes – please send me an invitation to complete the follow-up survey in three months.**

○ **No – please do not send me an invitation to complete the follow-up survey in three months.**

Now, please let us know what you think of this Final Report by clicking on the "Next" button.

Back | Next

CheckYourDrinking.net is for educational purposes only and is not to replace the advice of a healthcare professional. Copyright 2001-5 by V-CC. All Rights Reserved. All worldwide patent rights reserved.

File Name: Survey 1 Final Report Survey

Please remember that this survey is completely anonymous, and your voluntary feedback is very much appreciated. Thanks again for helping us out.

1. How useful did you find the Final Report feedback?

- ◯ Not at all useful
- ◯ Slightly useful
- ◯ Somewhat useful
- ◯ Extremely useful

2. Was any of the information surprising to you?

- ◯ No, the information was not surprising
- ◯ It was surprising how much more I drink that other people
- ◯ It was surprising how much less I drink that other people
- ◯ I found something else surprising. Please explain what you found surprising in the space below:

3. Is there any other information that you would like to see, or do you have any suggestions for how we can improve the information in the Final Report?

4. There were three graphs on the feedback report that compared your drinking to others in the general population (see examples below). Please put a check box underneath the graphs that you found most useful (If you didn't find any of the graphs useful, please don't check any of them).

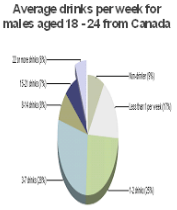

☐

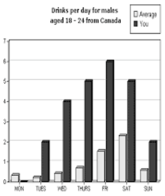

☐

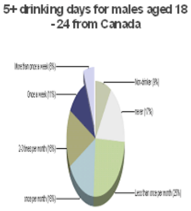

☐

5. If you submitted a description of your own drinking, did the feedback seem to capture the amount you actually drink?

- ◯ Yes, it probably did
- ◯ No, I usually drink less than once a week
- ◯ No, my drinking varies over time

6. Using a scale from 0 to 10, where 0 means "no risk" and 10 means "high risk," check the number that indicates to what extent you believe that you would personally be at risk of getting hurt or sick because of your own drinking?

☐	☐	☐	☐	☐	☐	☐	☐	☐	☐	☐
0	1	2	3	4	5	6	7	8	9	10
no risk		somewhat of a risk			medium risk			high risk		extremely high risk

7. Do you have any other comments that you would help us improve the Final Report?

If you'd like us to email you a copy of the published results from this survey, please check this box: ☐

Thank you very much for agreeing to participate in this survey. Please "Submit" to end the survey.

Submit

CheckYourDrinking.net is for educational purposes only and is not to replace the advice of a healthcare professional. Copyright 2001-5 by V-CC. All Rights Reserved. All worldwide patent rights reserved.
