# Supplementary material for: Formative Evaluation and Three-Month Follow-Up of an Online Personalized Assessment Feedback Intervention for Problem Drinkers
Source: J Med Internet Res. 2006 Apr 12;8(2):e5. doi: 10.2196/jmir.8.2.e5 (PMC1550700; doi:10.2196/jmir.8.2.e5)
Supplement: Supplementary file 2 [file jmir_v8i2e5_app2.pdf]

Dear Iroc

Three months ago you gave us your feedback on the Check Your Drinking survey, a new tool we are developing to help people evaluate their drinking. You gave us your e-mail address at that time so we could contact you and ask how you're doing. You can help us help others by answering a few brief questions about your experience.

Your feedback will help us understand how you feel about our program, and if it has changed how you think about drinking in any way. It's important for us to receive your feedback so we can make the program better.

We have a brief survey that we are hoping you will complete. To access the survey please click on the link below (If the below link is displayed on two lines please copy and paste the full link into your browser):

http://www.checkyourdrinking.net/survey/SS1.aspx?id=MTYwMjA=-lwQeMGzK/YY=

Your answers to the questionnaire will be confidential and anonymous. Your responses will be combined with the answers of all the other people who respond and we will not identify you or record any of your personal information.

Thanks for helping us help others!

Sincerely,

The Stop Smoking Center Support Team

And

The AHC Support Team

CHECK YOUR
DRINKING

## Three Month Follow-up to the Check Your Drinking Survey

Thanks for agreeing to help develop our new Check Your Drinking survey. We will ask you about your experiences with drinking over the past three months. We will also ask about how drinking and smoking go together for you because this is an important link as people work to improve their health.

When you fill in these questions, please think about the last three months.

1. At the present time, do you smoke cigarettes daily, occassionally, or not at all?

- ○ Daily
- ○ Occasionally
- ○ Not at all

2. When you drink alcohol, do you ever experience a strong urge, desire or thoughts about smoking?

- ○ Don't drink alcohol
- ○ Never
- ○ Occassionally
- ○ Frequently
- ○ All the time

3. Within the last three (3) months, to what extent did you believe that you would personally be at risk of getting hurt or getting sick because of your drinking?

Using a scale from 1 to 10, where 1 means "no risk" and 10 means "high risk", check the number that indicates to what extent you believed that you would personally be at risk of getting hurt or getting sick because of your own drinking?

| | 1 | 2 | 3 | 4 | 5 | 6 | 7 | 8 | 9 | 10 | |
|---|---|---|---|---|---|---|---|---|---|---|---|
| No risk | ○ | ○ | ○ | ○ | ○ | ○ | ○ | ○ | ○ | ○ | High risk |

It would be very helpful for us if you would fill in the Check Your Drinking Screener for us again, this time thinking about your drinking over the last three months.

Back   Next

CheckYourDrinking.net is for educational purposes only and is not to replace the advice of a healthcare professional.
Copyright 2001-5 by V-CC. All Rights Reserved. All worldwide patent rights reserved.

CHECK YOUR DRINKING

You are:

- female
- male

Your date of birth: ---------- -------------------- ---

How much do you weigh? ------

How much would you say each drink costs you: -- example: 3.75

You are taking this test:

- For yourself
- For someone you know

Back Next

CheckYourDrinking.net is for educational purposes only and is not to replace the advice of a healthcare professional.
Copyright 2001-5 by V-CC. All Rights Reserved. All worldwide patent rights reserved.

CHECK YOUR DRINKING

One Standard Drink Equals...

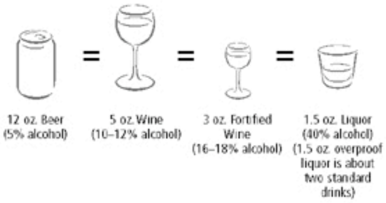


1. Within the last three (3) months, how often did you have a drink that contains alcohol?

- ◯ never
- ◯ monthly or less
- ◯ 2-4 times a month
- ◯ 2-3 times a week
- ◯ 4 or more times a week

2. Within the last three (3) months, on a typical day when you did drink, how many drinks containing alcohol did you have?

- ◯ 0-2
- ◯ 3-4
- ◯ 5-6
- ◯ 7-9
- ◯ 10+

3. Within the last three (3) months, how often did you have 5 (five) or more drinks on one occassion?

- ◯ never
- ◯ less than monthly
- ◯ once per month
- ◯ 2-3 times per month
- ◯ weekly
- ◯ 2-4 times per week
- ◯ daily or almost daily

4. How often during the last three (3) months have you found that you weren't able to stop drinking once you started?

- ◯ never
- ◯ less than monthly
- ◯ monthly
- ◯ weekly
- ◯ daily or almost daily

5. How often during the last three (3) months have you failed to do what's normally expected from you because of drinking?

- ◯ never
- ◯ less than monthly
- ◯ monthly
- ◯ weekly
- ◯ daily or almost daily

Back Next

CheckYourDrinking.net is for educational purposes only and is not to replace the advice of a healthcare professional.
Copyright 2001-5 by V-CC. All Rights Reserved. All worldwide patent rights reserved.

6. How often during the last three (3) months have you needed a first drink in the morning to "get yourself going" after a heavy drinking session?

- never
- less than monthly
- monthly
- weekly
- daily or almost daily

7. How often during the last three (3) months have you had a feeling of guilt or remorse after drinking?

- never
- less than monthly
- monthly
- weekly
- daily or almost daily

8. How often during the last three (3) months have you been unable to remember what happened the night before because you had been drinking?

- never
- less than monthly
- monthly
- weekly
- daily or almost daily

9. Have you or someone else been injured as a result of your drinking?

- no
- yes, but not within the last three (3) months
- yes, during the last three (3) months

10. Has a relative or friend or doctor or other health professional ever shown concern about your drinking, or suggested that you cut down?

- no
- yes, but not within the last three (3) months
- yes, during the last three (3) months

Back Next

CheckYourDrinking.net is for educational purposes only and is not to replace the advice of a healthcare professional.
Copyright 2001-5 by V-CC. All Rights Reserved. All worldwide patent rights reserved.

CHECK YOUR DRINKING

One Standard Drink Equals...

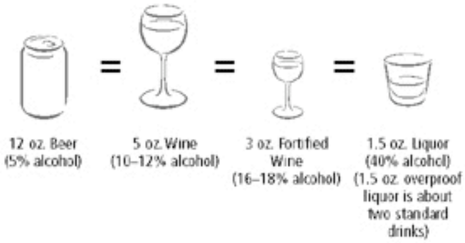


11. What was your drinking like during a typical week in the last three (3) months? We realize that this will only be a rough estimate, but please indicate the approximate number of drinks you usually drank on each day of the week.

| MON | TUES | WED | THURS | FRI | SAT | SUN |
|---|---|---|---|---|---|---|
| --- | --- | --- | --- | --- | --- | --- |

12. What is the greatest number of drinks you've had on one day in the past three (3) months?

---

Back Next

CheckYourDrinking.net is for educational purposes only and is not to replace the advice of a healthcare professional.
Copyright 2001-5 by V-CC. All Rights Reserved. All worldwide patent rights reserved.

CHECK YOUR DRINKING

13. Within the last 3 months was there ever a time that you felt your alcohol use had a harmful effect on your friendships or social life?

◯ no

◯ yes

14. Within the last three (3) months was there ever a time that you felt your alcohol use had a harmful effect on your physical health?

◯ no

◯ yes

15. Within the last three (3) months was there ever a time that you felt your alcohol use had a harmful effect on your outlook on life (happiness)?

◯ no

◯ yes

16. Within the last three (3) months was there ever a time that you felt your alcohol use had a harmful effect on your home life or marriage?

◯ no

◯ yes

17. Within the last three (3) months was there ever a time that you felt your alcohol use had a harmful effect on your work, studies or employment opportunities?

◯ no

◯ yes

18. Within the last three (3) months was there ever a time that you felt your alcohol use had a harmful effect on your financial position?

◯ no

◯ yes

Back Next

CheckYourDrinking.net is for educational purposes only and is not to replace the advice of a healthcare professional.
Copyright 2001-5 by V-CC. All Rights Reserved. All worldwide patent rights reserved.

Do you have any additional comments?

Thank you very much for agreeing to participate in this survey. If you are interested in our full Alcohol Reduction software program, please click here .

Click "Submit" to end the survey.

Back Submit

CheckYourDrinking.net is for educational purposes only and is not to replace the advice of a healthcare professional.
Copyright 2001-5 by V-CC. All Rights Reserved. All worldwide patent rights reserved.
