# Supplementary material for: Formative Evaluation and Three-Month Follow-Up of an Online Personalized Assessment Feedback Intervention for Problem Drinkers
Source: J Med Internet Res. 2006 Apr 12;8(2):e5. doi: 10.2196/jmir.8.2.e5 (PMC1550700; doi:10.2196/jmir.8.2.e5)
Supplement: Supplementary file 3 [file jmir_v8i2e5_app3.pdf]

Print printer friendly version of Final Report

Email email this report to yourself or your physician

Final Report For Sample

The average number of drinks you reported consuming per week was 14.

How do you compare to males your age from Canada? The highlighted slice of the pie chart below is where your drinking fits compares to other males in your age range from Canada.

Average drinks per week for males aged 25 - 34 from Canada

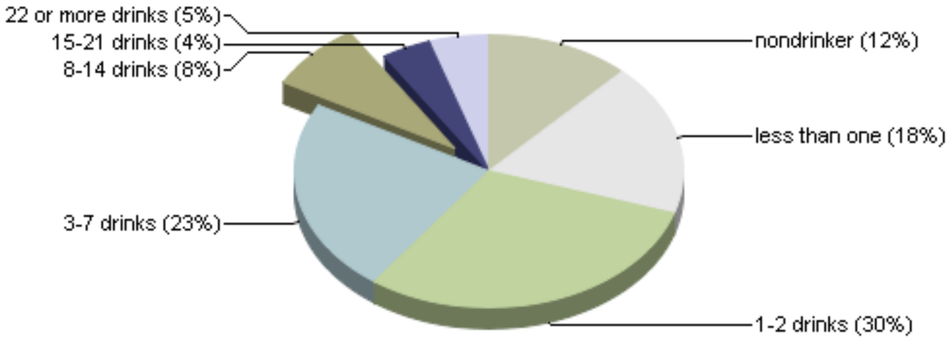

Within the last twelve (12) months:

- You reported drinking on approximately 71.2% of days in the last year.
- You reported that you drank a total of 728 drinks in the last year.

This also means that:

- You spent approximately $2184 in the last year, depending on where you drank (at home, in a bar, etc.).
- You consumed (on average) 200 calories from alcohol on days that you drink. Based on the total amount of drinking you had enough alcohol to add roughly 21 pounds to your weight in the last year. **Note:** One drink has about 100 calories and 3,500 calories roughly equals 1 extra pound of weight.
- You also reported that within the past year, the greatest number of drinks you had on one occasion was 10 drinks.

Your Drinking Patterns

The following graph outlines how your weekly alcohol consumption rates compare to other males in your age range from Canada.

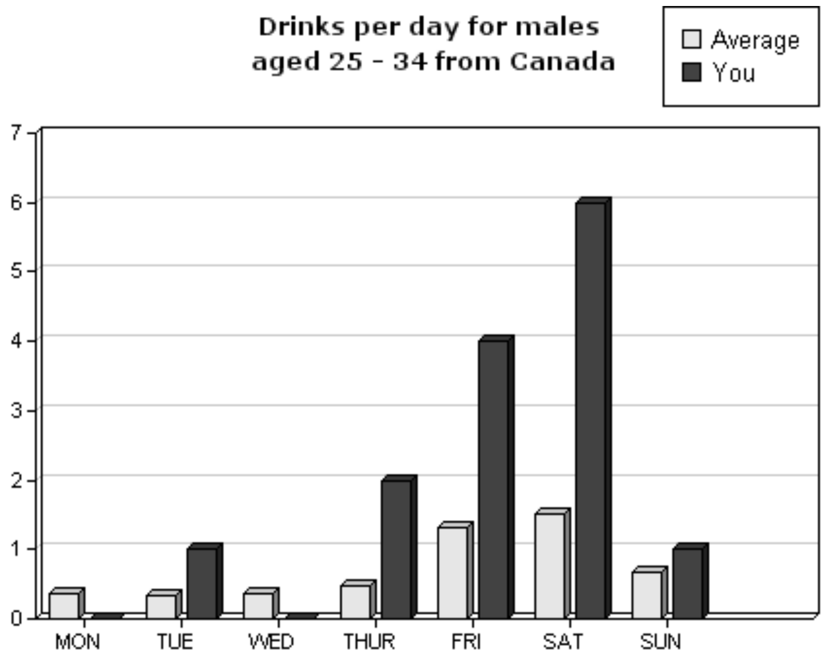

Heavy Drinking Days

How often do males from Canada in your age range drink five or more drinks on one occasion? The highlighted slice shows where your drinking fits into the chart:

5+ drinking days for males aged 25 - 34 from Canada

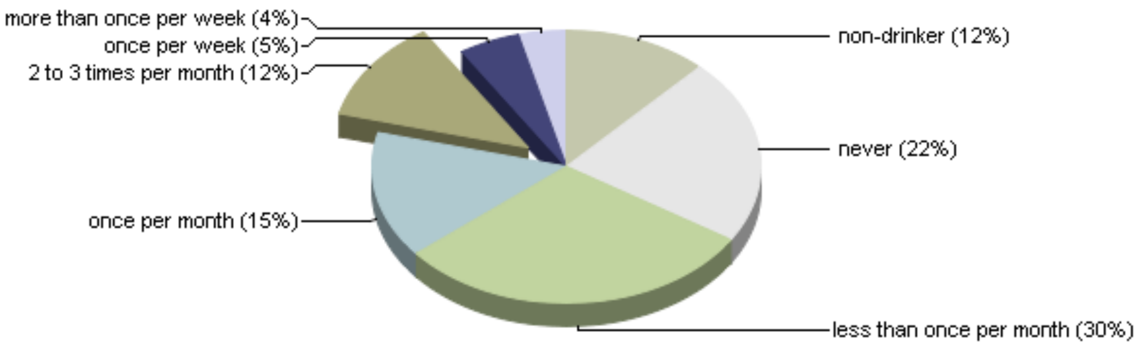

Why care about having five or more drinks on the same day? Heavy drinking days place you at increased risk of experiencing negative consequences because of your drinking. If you got rid of these heavy drinking days you would reduce your chances of experiencing problems by about 50%.

Alcohol-Related Consequences

In the last twelve (12) months you reported your drinking had a harmful effect on your:

- friendships or social life
- physical health

- level of happiness

Risky Drinking

A national survey looked at how much people drank in a week and how their drinking might be affecting different areas of their lives. People were asked about their physical health, outlook on life, friends/social life, relationships with their spouse, partner or children, home life, financial position and work or studies. The results showed that the more people drank in a week, the greater the chance that drinking was affecting more and more areas of their lives.

How likely are you to have problems as a result of your drinking? The white bar on the chart below shows where you fit:

Chance of negative consequences related to number of drinks per week

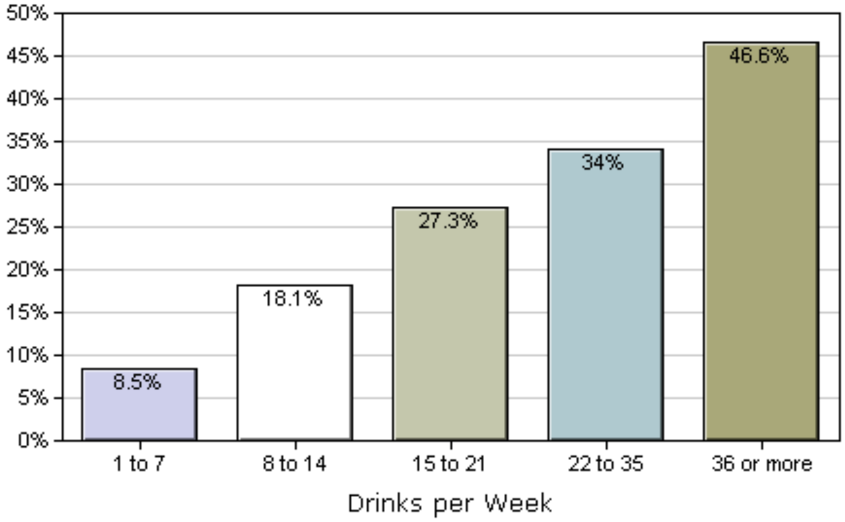

AUDIT Score

The AUDIT questionnaire was developed by the World Health Organisation to evaluate a person’s use of alcohol. The AUDIT score shows whether a person’s drinking should be considered a problem. Higher scores usually mean serious problems. The below chart is in the shape of a pyramid to show that there are more people with low AUDIT scores than high ones.

Your AUDIT score is 11. Based on the answers that you supplied during the CYD survey, the white area of the chart shows where your score falls.

Audit score (risk of alcohol-related harm)

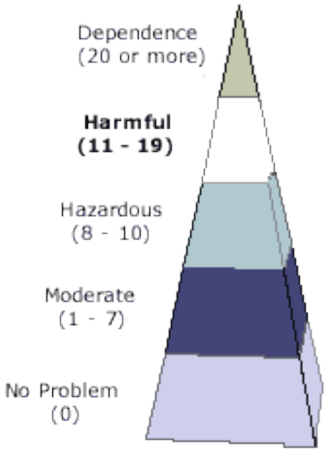

The following describe the meaning of AUDIT scores

Score of	Means
0-7	Low risk of alcohol-related harm
8-10	High risk of experiencing alcohol-related harm (some people in this range will already be experiencing significant harm)
11-19	A person scoring in this range will already be experiencing significant alcohol-related harm.
20+	A person scoring in this range may be alcohol dependent and is advised to see a health care professional about their drinking.

Burning Alcohol

Your liver metabolizes (or burns) alcohol at a constant rate – about 1 gram per hour for every 10 kilograms (22 pounds) of your body weight. Exercising or drinking coffee will not get the alcohol out of your body any sooner.

Although you may not feel some of the effects of alcohol your body is working long after you drink to get rid of the alcohol in your system. Depending on how much you drink and weigh, your liver can be under extra strain for a very long time. This is one example of the health risks of heavy drinking.

You reported that you weigh 155 lbs. This means that:

- If your liver is healthy, burning one drink takes you 2 hours. Burning four drinks takes you about 8 hours. If you had ten drinks, it will take about 20 hours until there is no alcohol in your system.
- In the last year you spent about 1,447 hours under the influence of alcohol (based on your typical drinking during one week).

There are also other kinds of risk that heavy drinking presents. Even small amounts of alcohol can affect your ability to drive or operate heavy equipment safely. **If you have a drink, don't drive!** Take a bus or taxi, or get a lift from a friend who hasn't been drinking.

Sensible Drinking

Guidelines supported by the Centre for Addiction and Mental Health suggest that most people can drink up to two drinks a day without significant risk to their health, in the short or the long term.

Most people can and do drink safely and sensibly. This means no more than two drinks a day with a weekly maximum of 14 drinks for men and 9 drinks for women. It is also a good idea to make sure there are days when you don't drink at all. For some people, even 1 to 2 drinks per day would be too many. Pregnant women, for example, are advised to abstain from alcohol completely because even small amounts of alcohol could increase the risk to the unborn child. Heavy drinking days are especially dangerous to the unborn child. Certain health problems such as heart disease or cancer can make even moderate drinking unsafe.

Health Effects of Alcohol

We've included the following information in the event that you would like to learn more about how the use of alcohol can affect your health.

Your liver
Because the liver receives blood directly from the intestines, it takes the brunt of high alcohol concentrations. Heavy alcohol consumption can lead to two serious types of liver injury: *hepatic inflammation* (alcohol hepatitis) and *progressive liver scarring* (fibrosis or cirrhosis). (*Chedid et al 1991; Dufour et al 1993*)

Women are more at risk to serious alcohol-related liver injury; they develop cirrhosis at a lower dose of alcohol than men do. (*Marbet et al 1987*)

Heavy drinkers are 3 times more likely to develop liver cancer than non-drinkers. (*English & Holman, 1995*)

Your throat, stomach, and intestines
Alcohol is a cause of long-term throat inflammation that sometimes leads to cancer.
Inflammation occurs in part because alcohol reduces contraction of the smooth muscle in the lower throat. (*Keshavarzian et al 1994*)

People who drink more that 21 drinks per week have almost a ten-fold higher risk of throat cancer than those who consume fewer than 7 drinks per week. (*Vaughan et al 1995*)

Mouth cancers are six times more common in heavy alcohol users than in non-alcohol users.
(*American Cancer Society, 2002*)

Your pancreas
The pancreas is a gland behind your stomach that releases chemicals important for digesting food. Heavy alcohol use can lead to long-term pancreatic inflammation, weakening, and scarring. (*Haber et al 1995*)

Alcohol can also cause acute pancreatitis, a severe and very painful inflammation of the pancreas.

Your heart and circulatory system
The potential health benefits of moderate drinking (up to 2 drinks per occasion) do not apply to younger people, whose risk for heart disease is ordinarily very low.

Long-term drinking of more than four drinks per occasion has been linked to a variety of damaging effects on the heart and circulatory system. (*Davidson, 1989*)

Your brain
Alcohol can cause direct or indirect damage to nervous tissues. Long-term heavy drinking is linked to brain damage and

poor mental functioning. (*Spreen and Strauss, 1991*)

Your endocrine system (hormone regulation)
Alcohol interferes with the hormone regulation of a number of bodily activities. Men who have a history of heavy drinking often have lower levels of testosterone and increases in female sex steroids, such as estradiol and estrone.

Emergency Department
Patients treated in an emergency department for an unintentional injury are 13.5 times more likely to have drunk 5 or more alcohol-containing drinks within 6 hours of their injury. Alcohol-related unintentional injuries and deaths include motor vehicle crashes, drowning, falls, hypothermia, burns, suicides, and homicides. (*Vinson, 2003*)

Road traffic crashes
Road traffic crashes are the leading cause of death for people aged 1-34 years. They are also a leading cause of hospitalization for serious injury. Alcohol is involved in around 40% of crashes (LTSA, 2000; NHTSA, 2003).

Compared with a person with no alcohol in their blood, a person with a blood alcohol concentration (BAC) of 0.10% is between 13 and 18 times more likely to have any crash and 50-90 times more likely to have a fatal crash. (*Miller, 2001*)

Unlike what some people believe, vehicle occupants with high levels of alcohol in their system (high BAC) are more likely to be seriously injured or to die in the event of a crash. (*Soderstrom, 1993*)

Alcohol and Violence
In 1997, about 40% of all crimes (violent and non-violent) were committed by people who had alcohol in their system. (*Bureau of Justice Statistics, 1998*)

In 1997, 40% of convicted rape and sexual assault offenders said that they were drinking at the time of their crime. (*Greenfield, 2000*)

Approximately 72% of rapes reported on college campuses occur when victims are so intoxicated they are unable to consent or refuse. (*Wecshler, 2004*)

Reducing Your Risk

There are many things that you can do to reduce the risk of hurting yourself or others. Here are some small steps you can take to start making a change:

- Don’t drink in any situation where there’s a risk of accident or injury — for example, drinking and driving.
- Don’t mix alcohol with other drugs — especially other depressants like tranquilizers, barbiturates, heroin or other opioids.
- Try to reduce by one or two days the number of days you drink each week.
- Decide how much you will drink ahead of time and keep yourself to this limit.
- Take a limited amount of money with you if you go out to have a drink.
- Keep track of the amount you drink.
- Alternate alcoholic with non-alcoholic beverages when you drink.
- Choose alcoholic beverages with lower alcohol content.
- If you are out drinking with friends, make sure at least one person stays sober. If that person is driving they should not drink any alcohol at all.
- Do not become intoxicated with people you do not know and trust; criminal victimization is a much greater risk when you are drunk.
- Remember the need to practice safer sex — always use condoms.

Click here to log out

HOME | ABOUT | PRIVACY POLICY | LEGAL INFORMATION | FAQ | FEEDBACK

CheckYourDrinking.net is for educational purposes only and is not to replace the advice of a healthcare professional.
Copyright 2001-5 by V-CC. All Rights Reserved. All worldwide patent rights reserved.
